# Blinded Minds: The Role of Cataracts in Cognitive Decline and Dementia

**DOI:** 10.7759/cureus.89166

**Published:** 2025-07-31

**Authors:** Amir A Estil-las, Martena Grace, Camelia Arsene, Jessie Gonzalez La Rosa

**Affiliations:** 1 Medical School, Ross University School of Medicine, Miramar, USA; 2 Internal Medicine, Trinity Health Oakland, Pontiac, USA; 3 Psychiatry, Larkin Community Hospital, Miami, USA

**Keywords:** alzheimer's disease, cataracts, cataract surgery, dementia, mild cognitive impairment (mci), pathophysiology, visual impairment

## Abstract

Dementia and cataracts are two leading causes of disability among the elderly population throughout the world. The relationship between both pathologies remains underrecognized. This paper explores the emerging evidence linking cataracts to cognitive decline and dementia, proposing that visual impairment may contribute to neurodegeneration. Popular hypotheses supporting these claims include circadian rhythm disruption, sensory deprivation, reduced social engagement, and increased cognitive load. Epidemiological data supports the co-prevalence of cataracts and dementia. Patients undergoing cataract removal have shown improved performance on cognitive assessments, suggesting that vision correction may enhance neural efficiency and quality of life; moreover, interventional studies demonstrate that cataract surgery not only improves visual impairment but may also slow cognitive deterioration. This paper also elucidates the clinical and public health implications of the integration of vision care into cognitive health strategies. The global dementia burden is projected to rise sharply in the coming decades; we propose that early intervention through cataract surgery may offer a cost-effective method to preserve cognitive function. Ultimately, identifying and treating visual impairment may serve as a crucial step toward delaying the progression of dementing illnesses.

## Introduction and background

Introduction

Cataracts are a disease process that involves progressive opacification of the lens, obstructing light, and ultimately contributing to blindness [1,2]. Multiple etiologies are attributed to the formation of cataracts; however, most of them are due to degenerative processes. The proteins found within the lens denature and coagulate, leading to diminished light passing through the lens [2,3]. Cataracts can additionally be further classified by the mechanism responsible for the degenerative changes. Some common variants include congenital, senile, subcapsular, nuclear, sclerotic, cortical, Christmas tree, and trauma-induced [2]. Regardless of the etiology or mechanism behind its formation, the treatment is surgical intervention [4]. Cataract surgery is one of the most successful clinical interventions, with a reported 95% improvement in activities of daily living, visual acuity, and decreased mortality [4]. If left untreated, cataracts may go on to develop secondary complications like glaucoma and uveitis, thus leading to a more complex clinical management with less secure outcomes.

In 1990, it was estimated that about 37 million people worldwide were blind, with an increase of 1-2 million cases every year. Of these cases, 75% were caused by preventable etiologies, and 40% of all cases were attributed to the formation of cataracts [5]. In a 2020 systematic review, Hashemi et al. reported a 17.20% prevalence of any cataract in 45 studies with a sample size of 161,947, of which the majority were from the Office for Western Pacific Region [6]. Demonstrating that cataracts remain a pathological burden to healthcare in modern times [7,8].

Dementia is the seventh leading cause of death and is a major factor contributing to dependency and disability throughout the world [9]. Approximately 47 million people live with dementia worldwide, and it is estimated that by the year 2050, 131 million people will be affected [8]. The known risk factors responsible for contributing to dementia include smoking, alcohol use, atherosclerosis, plasma homocysteine, Down syndrome, genetic influence, and diabetes [10]. Interestingly, however, individuals with visual impairment have been shown to have an increased risk for developing dementia [11]. The precise mechanism behind this interesting correlation remains unknown; however, some theories for its co-prevalence include common risk factors, Beta-amyloid deposits, complement factor H, and apolipoprotein E genetic variants [11].

In this literature review, we aim to bring forth the current scientific theories behind the association between cataracts and dementia. We will explore common risk factors, potential genetic variants, and certain lifestyle factors that may contribute to this correlation.

## Review

Overview of cataracts and dementia

Cataracts: Definition, Etiology, and Risk Factors

**Figure 1:**
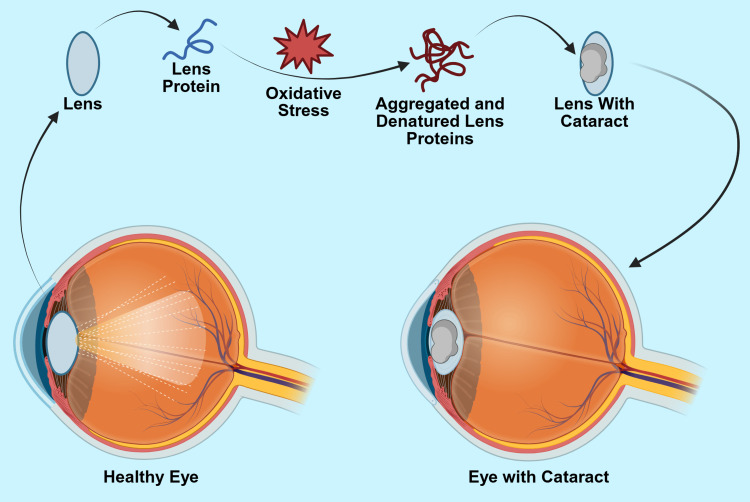
Pathophysiology of Cataract Formation Image Credits: Amir A. Estil-las and Martena Grace Created in BioRender. Estil-las, A. (2025) https://BioRender.com/l70ewh6

The lens of the eye is a transparent biconvex structure that aids in the focusing and transmission of light through the eye. It is composed of regularly arranged fibers, encapsulated by a thin capsule. The lens fibers originate from the anterior epithelium, to which they migrate from the periphery of the lens toward the center [12]. Cataracts refer to the opacification of the lens structure, which interferes with light transmission, ultimately impairing vision and potentially leading to blindness. While cataracts can develop through various mechanisms, oxidative stress is the predominant contributor to lens opacification (Figure 1) [12]. 

As stated previously, cataracts arise from multiple etiologies and mechanisms. Risk factors associated with cataract formation include genetic predisposition, aging, trauma, nutritional deficiencies, metabolic disorders, and medication use. Congenital cataracts have been associated with genetic mutations, maternal and fetal environmental influences, as well as racial and ethnic factors. Approximately 33% of congenital cataracts are hereditary [13]. Genetic disorders like Lowe syndrome and neurofibromatosis are some of the many conditions attributed to congenital cataracts [13]. Maternal malnutrition and exposure to infections like rubella, toxoplasmosis, and cytomegalovirus during pregnancy are known contributing factors to congenital cataracts [13]. Teratogenic agents such as alcohol, corticosteroids, and ionizing radiation are also associated with congenital cataract formation. Moreover, epidemiological studies have shown that women, African American, and Hispanic American populations have nearly twice the risk of developing cataracts when compared to Caucasian populations [13].

Age-related cataracts (senile cataracts) are defined as lens opacities that occur in adults over the age of 50, with no attributable traumatic, chemical, or radiation-induced cause [13]. Senile cataracts have been attributed to several risk factors and causes; A study by Minassian et al. revealed that recurrent diarrhea and dehydration crises were associated with 36% of senile cataracts in the study [13,14]. Smoking has been associated with a two-to-threefold increase in cataract incidence, most likely due to aromatic compounds in cigarette smoke interacting with the lens proteins, leading to opacification [13,15-17]. Hypertension, particularly in patients with comorbid diabetes mellitus, was found to increase cataract risk [13]. Alterations in lipid metabolism, like those found in Smith-Lemli-Opitz syndrome, mevalonic aciduria, and cerebrotendinous xanthomatosis, have also been associated with cataract development [13].

Traumatic cataracts occur after direct injury to the eye, disrupting either the lens or the lens capsule. The injury leads to eventual denaturation of the lens proteins and opacification [13].

Disorders that alter metabolism, such as galactosemia, diabetes mellitus, hypocalcemia, hypothyroidism, copper metabolism errors, and micronutrient deficiencies, have also been implicated in increasing cataract risk.

Various pharmacological agents have been linked to increasing the risk of cataract development; some include corticosteroids, tranquilizers, radiomimetic drugs, methotrexate, oral contraceptives, ergot derivatives, miotics, sulfanilamides, streptozotocin, methoxsalen, isotretinoin, epinephrine, psoralen, and thiazide diuretics [13]. Moreover, prolonged use of hormone replacement therapy and excessive alcohol consumption have also been attributed to an increased risk [13].

Ultraviolet (UV) radiation is a major component of cataractogenesis. Hollows and Moran found that regions in Australia with high UV exposure had higher prevalence rates of cataracts [18]. Cataracts are more likely to form under any exposure to the UV radiation spectrum, but UV-B radiation has been shown to cause more significant damage to the lens than UV-A [19].

Dementia: Definition, Types, and Risk Factors

Dementia is a clinical syndrome marked by a decline in several domains of cognitive function. Any decline in memory, language, attention, visuospatial ability, and executive functioning that is severe enough to interfere with the ability to perform independent daily activities is classified as dementia [8]. The diagnosis is multidisciplinary in nature, often involving detailed history-taking, cognitive assessments like the Mini-Mental State Examination (MMSE) or Montreal Cognitive Assessment (MoCA), neuropsychological testing, and brain imaging [8].

The degree of dementia exists on a spectrum - individuals may experience varying severities of dementia, often dictated by the MMSE. Some individuals may suffer from cognitive decline without any impairment in the ability to perform daily activities, which is classified as mild cognitive impairment (MCI). MCI often precedes the development of full dementia.

**Figure 2:**
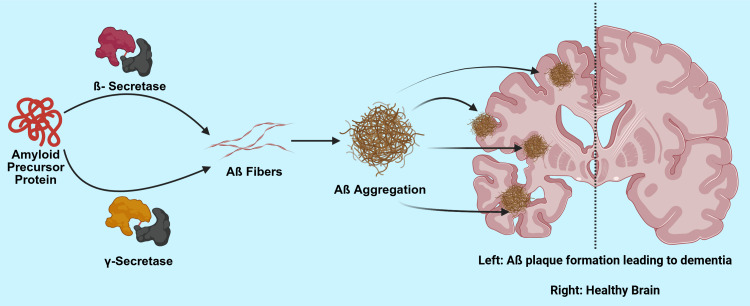
Pathophysiology of Alzheimer’s Dementia Image Credits: Amir A. Estil-las and Martena Grace Created in BioRender. Estil-las, A. (2025) https://BioRender.com/ww8ze1i

Like cataracts, there are multiple subtypes of dementia, each characterized by unique features and pathologies. Of all subtypes of dementia, the most common form is Alzheimer’s disease. Alzheimer’s disease is marked by memory loss due to amyloid-beta/tau pathology (Figure 2) [20]. Vascular dementia results from cerebrovascular injury, which results in a stepwise clinical cognitive decline [21]. Dementia with Lewy bodies presents with fluctuating cognition and the presence of visual hallucinations [22]. Frontotemporal dementia predominantly begins by causing a decline in behavior and language. Parkinson’s dementia arises in the late stages of Parkinson's disease. Mixed dementia is defined as dementia that involves several features of more than one dementia [23]. 

The risk factors of dementia vary depending on the subtype of dementia; however, aging, hypertension, diabetes mellitus, hyperlipidemia, smoking, atrial fibrillation, elevated homocysteine levels, and genetic predispositions are shared risk factors among all dementias [24]. While most dementias are progressive in nature and are currently irreversible, early identification and management can improve quality of life and slow cognitive deterioration.

Alzheimer’s disease has become a focal point of scientific research in recent years. Alzheimer’s disease has increased in prevalence and is a major contributor to decreased quality of life across the elderly population. The United States federal government has significantly increased funding for Alzheimer’s disease and dementia research. In the fiscal year 2024, the National Institutes of Health allocated approximately $3.8 billion to Alzheimer’s and related dementia research, a substantial rise from the $448 million invested annually during the initial enactment of the National Alzheimer’s project [25].

Recent advancements in Alzheimer’s treatment include the Food and Drug Administration’s approval of lecanemab, a monoclonal antibody therapy targeting amyloid-beta plaques in brain tissue [26]. Clinical trials have demonstrated that lecanemab can slow cognitive and functional decline in individuals with early-stage Alzheimer’s disease by approximately 27% in a period of 18 months [27]. While lecanemab is not a cure for Alzheimer’s disease, it represents a significant step forward in modifying disease progression, offering patients and caregivers hope for extended quality of life [28]. Current ongoing research continues to explore the long-term efficacy and safety of lecanemab, as well as the development of additional therapeutic options.

As the population ages, deficits such as visual impairment and cataracts are becoming increasingly recognized not just as quality of life concerns, but as contributors to cognitive decline. Growing evidence suggests that visual impairment may exacerbate or accelerate neurocognitive deterioration, mimicking or inducing dementia.

Evidence for association between visual impairment and cognitive decline

The current literature indicates that there may be a causative association between cataracts and dementia. “A 2023 meta-analysis by Xiong et al. analyzed 13 studies comprising 798,694 participants and found an increased risk of developing all-cause dementia, with a pooled hazard ratio (HR) of 1.22 (95% CI: 1.08-1.38)” [29]. The study further stratified the risk of developing subtypes of dementia. Participants with cataracts had an increased risk of developing Alzheimer’s disease with a pooled HR of 1.18 (95%CI: 1.07-1.30); Vascular dementia had a pooled HR of 1.21 (95%CI: 1.02-1.43); and MCI had a pooled HR of 1.30 (95%CI: 1.13-1.50) [29].

Additionally, a 2024 meta-analysis on the risk of dementia or cognitive impairment in patients with cataracts, performed by Wang et al., demonstrated similar results to the Xiong et al. study. Wang et al. analyzed 11 publications, consisting of 489,211 total participants. The results showed an association between cataracts and cognitive impairment, with an odds ratio (OR) of 1.32 (95% CI: 1.21-1.43) [30]. Furthermore, the presence of cataracts was associated with a higher risk of all-cause dementia with a relative risk (RR) of 1.17 (95%CI: 1.08-1.26) [30]. Sub-group analysis also identified an increased risk of developing Alzheimer's disease and vascular dementia with an HR of 1.28 and 1.35, respectively.

Despite the epidemiological link between cataracts and dementia, biological and psychosocial analyses are scarce. The following section will examine the leading hypotheses proposed in current literature, exploring various mechanisms of action thought to be responsible for linking cataracts and dementia.

Proposed mechanisms linking cataracts and dementia

Circadian Rhythm Disruption

It has been proposed that sleep and circadian rhythm disruptions are a risk factor for developing dementia, particularly Alzheimer’s disease. Upon having sleep disturbances, neuronal activity, oxidative stress, and tau phosphorylation all increase, which is what is thought to contribute to synaptic activity via amyloid-beta and tau aggregation - thus leading to Alzheimer’s disease [31]. Additionally, sleep deprivation impairs the glymphatic and lymphatic clearance systems responsible for removing macromolecules from the brain, thereby contributing to increased amyloid-beta and tau protein burden [31,32].

The human circadian rhythm is modulated by the Suprachiasmatic nucleus (SCN). The SCN indirectly controls the amount of melatonin the pineal gland secretes via multisynaptic pathways [33]. Another major modulator of the sleep-wake cycle is the quantity of light, particularly blue light, that retinal photoreceptors receive throughout the day [33,34]. Upon photo stimulation, retinal photoreceptors interact with the SCN to prevent further secretion of melatonin, thus promoting a wakeful state. Eye pathologies that result in decreased light input, like cataracts, may disrupt circadian regulation and thereby contribute to the risk of developing Alzheimer’s disease [35].

The prevalence of sleep disorders is higher in elderly populations when compared to younger populations [36]. Foley et al. analyzed over 9,000 participants over the age of 65 and concluded that more than half reported symptoms of insomnia [36]. As previously discussed, cataracts are a leading cause of blindness and visual impairment, with senile cataract being the most common variant. It is postulated that as the lens nucleus hardens with age and accumulates proteins, there is decreased blue light reaching the retina [37]. These decreases in blue light may contribute to increased sleep disturbances and subsequently dementia [38]. Upon correction of cataracts via surgical intervention, multiple studies were performed to assess the improvement in sleep quality; however, although they mostly yielded positive results, there are multiple studies reporting that there was no statistical difference in sleep quality after surgical intervention. Per Asplund and Linbald’s study, there was a self-reported improvement in sleep quality 1 month after cataract surgery [39]. Alexander et al.’s study also further supports a positive correlation with cataract correction and quality of sleep, with over 900 patients reporting improvement at 1 month, 6 months, and 12 months postoperatively [40].

As one ages, the prevalence of systemic illness and degeneration increases, of which some pathologies may contribute to the degeneration of SCN neurons, altering the quality of sleep and circadian rhythm. Due to this theory, it is thought that early correction of cataracts may reduce the risk or slow the progression of dementing illness, due to the correction of sleep disturbance; however more studies are needed to determine the extent of contribution cataracts impose on sleep changes that result in dementing illnesses, and to what extent cognitive function improves following cataract correction [33].

Shared Pathophysiology

Despite cataracts and dementing illnesses being distinct clinical entities, growing evidence suggests that their link is due to sharing common risk factors and pathophysiological mechanisms. A meta-analysis by Xiong et al. demonstrated that oxidative stress and inflammatory pathways are strong potential contributors to the observed association between cataracts and dementia [29].

Oxidative stress refers to an imbalance between the production of reactive oxygen species (ROS) and the body’s ability to neutralize them via antioxidant defenses [41]. In the context of neurodegenerative diseases, including Alzheimer’s, Parkinson’s, and amyotrophic lateral sclerosis, excess ROS contributes to biomolecular damage within the central nervous system [41]. In Alzheimer’s disease, lipid peroxidation byproducts, caused by ROS, have been linked to neuronal toxicity and in promoting the aggregation of amyloid-beta proteins [41,42]. Chronic oxidative stress also induces neuroinflammatory cascades, further amplifying neuronal injury and accelerating disease progression.

Oxidative stress also plays a central role in cataract formation. The lens is particularly prone to oxidative stress due to lifelong exposure to UV radiation [43,44]. Over time, ORS accumulation leads to the modification and aggregation of crystallin proteins in the lens, leading to opacification and eventual cataract development [43]. Some potential treatments to protect the lens from oxidative damage include Nrf2 Activators, GSH enhancers, and renin-angiotensin-aldosterone system (RAAS) modulators; however, they have varying efficacy in preventing cataracts [43].

While the presence of oxidative stress and inflammatory changes in both dementia and cataracts is well-documented, the directionality and causality of their relationship remain uncertain. It is currently unclear whether cataract-related oxidative changes independently contribute to neurodegenerative risk or simply reflect systemic aging processes. Nevertheless, identifying these shared mechanisms offers a compelling area for further research, particularly in the context of early screening, risk stratification, and potential therapeutic interventions.

Although both oxidative stressors and inflammatory changes contribute to dementing illnesses and cataracts, more research is necessary to examine if oxidative stress alone is a risk factor for developing dementia in patients with existing cataracts.

Sensory Deprivation

Two prevailing theories in the domain of sensory deprivation may explain the association between sensory loss and cognitive decline: the cascade theory and the common cause theory [45]. The cascade theory states that upon having an impaired sense, there is a strain to cognitive systems, leading to impaired cognitive performance [45-49]. The common cause theory states that sensory loss and cognitive impairment are related via cerebral atrophy and pathophysiological changes of the brain due to aging [50,51].

Per the principle of neuroplasticity, the brain compensates for sensory deficits by remodeling neuronal pathways, particularly in regions responsible for processing other sensory inputs. However, some studies suggest this remodeling may be maladaptive, resulting in disorganized or inefficient networks that ultimately contribute to cognitive decline [44,51-53]. Despite potential maladaptive changes occurring, studies have shown that individuals who undergo cataract corrective surgery experience slower rates of cognitive decline [44, 54-56]. Similar trends have been observed following other conditions that result in visual impairment, like glaucoma [44, 57-60]. These findings support the notion that visual impairment may be a modifiable risk factor for cognitive impairment and dementia.

It is difficult to firmly conclude that degenerating visual acuity alone is responsible for impacting cognitive function. Any decrease in a person’s sense has a multitude of multidomain changes in the affected person’s life: social isolation, greater dependence on others, and depression, which are known factors that contribute to the progression of dementing illnesses [45]. A study by Ge et al. analyzed the impact of loneliness and visual impairment on the rate of cognitive decline and concluded that patients with visual impairment and elevated levels of loneliness experienced a more deleterious impact on cognitive function than when compared to patients with isolated visual impairment [61,62].

The precise neurobiological mechanisms linking sensory deprivation to dementia remain uncertain, but the consistent correlation highlights the need for further research. Targeted screening and early interventions may offer new opportunities for reducing dementia risk in vulnerable populations, as it is one of the most burdensome illnesses of the current times [44].

Neurodegenerative Spillover Hypothesis

The neurodegenerative spillover hypothesis is a theory suggesting that amyloid-beta (Aβ), one of the hallmark proteins responsible for Alzheimer’s disease, may contribute to not only neurodegeneration but also to the pathogenesis of cataracts, implying that there is a shared molecular pathway involving Aβ aggregation.

Goldstein et al. examined postmortem eye and brain tissue from nine individuals with Alzheimer’s disease and eight matched controls without the disease. The researchers discovered electron-dense Aβ deposits within the cytoplasm of the supranuclear/deep cortical lens fibers only in the group with Alzheimer’s disease, indicating a possible disease-specific pattern of lens involvement [63].

Moncaster et al. produced a study further supporting the theory that Aβ contributes to cataract formation. The researchers explored the Aβ burden found within the lenses of individuals with Down syndrome. Evaluation of the lenses demonstrated the characteristic supranuclear opacification and supranuclear Aβ accumulation, just like the ones found in Alzheimer’s disease [64]. Down syndrome increases the risk of Alzheimer’s disease due to the triplication of chromosome 21, which contains the APP (amyloid precursor protein) gene [64]. The extra copy of chromosome 21 leads to excess APP production, eventually leading to an increased level of amyloid burden.

In 2022, Moncaster et al. analyzed transgenic mice with amyloid-producing genes and observed Aβ deposits within the lenses. They speculate that long-lived lens fibers, which have some capacity to break down and clear aggregated proteins, may accumulate Aβ before it appears in brain tissue, implying that Aβ buildup in the lens could precede cerebral amyloidopathy [65]. These findings were bolstered by a longitudinal comparison of Alzheimer’s disease-associated lens and brain traits, and bivariate genetic analyses from the Framingham Eye study participants [65,66]. Results from these studies may aid clinicians by indicating the need for ophthalmological evaluation or neuropsychological evaluation in individuals who are genetically predisposed to have amyloidopathies.

Comorbidities and Medications

Cataracts and cognitive impairment are both common age-related pathologies [67]. The prevalence of dementia and cataracts increases in adults over the age of 60 across North America, Western Europe, and the Western Pacific [67-69]. These conditions often coexist, and emerging research suggests they may share several modifiable and non-modifiable risk factors, potentially contributing to their comorbidity.

Advancing age, female gender, smoking, diabetes, hypertension, cardiovascular disease, obesity, lower socioeconomic status, and limited access to healthcare have all been identified as common risk factors for both dementia and cataracts [70-72]. As discussed previously, chronic systemic inflammation and oxidative stress are also central pathophysiological mechanisms underlying both diseases [73,74]. Furthermore, multimorbidity is a recognized predictor of both cognitive and visual decline, as individuals with a higher burden of chronic disease, such as diabetes and vascular disorders, are at a significantly greater risk for developing both cataracts and dementia [75].

Given the overlap in risk profiles, interventions targeting these shared risk factors, such as glycemic control, smoking cessation, and management of cardiovascular health, may play a role in reducing the incidence or severity of both conditions. These findings highlight the importance of a holistic, preventative approach in the care of aging populations.

Cataract surgery and cognitive outcomes

Recent research has highlighted a compelling link between cataract surgery and reduced cognitive decline. A study performed by Lee et al. found that individuals who underwent cataract extraction had a 29% lower risk of developing dementia, after adjusting for demographic and health-related confounders [76]. Similarly, a 13-year longitudinal analysis from the English Longitudinal Study of Ageing reported that cataract surgery was associated with a slower rate of cognitive decline, particularly in episodic memory [77]. A 2024 meta-analysis by Yeo et al. further reinforced these findings, showing a 25% reduced risk of cognitive decline across 24 studies totaling over 558,000 participants [78].

Moreover, cognitive improvements have been observed shortly after cataract surgery. Arai et al. conducted a prospective study showing enhanced cognitive test scores three months postoperatively in elderly patients, particularly those with MCI [79]. The Fujiwara-kyo Eye Study also found that prior cataract surgery significantly reduced the risk of MCI, suggesting that maintaining good visual acuity can positively affect cognitive health [80]. However, not all findings are uniformly positive; a cross-sectional study using Chinese CHARLS data showed an association between cataract surgery and lower cognitive scores, possibly reflecting the severity of visual impairment before surgery [81]. Additionally, factors like the timing of surgery, pre-existing neurodegeneration, socioeconomic disparities in access, and the presence of comorbidities may influence outcomes and require further investigation.

Despite promising findings, several limitations persist across the literature. Many studies are observational in nature, limiting causal inferences. Cognitive testing methodologies and surgical timing vary widely, making cross-study comparisons difficult. There is also limited data on how cataract surgery interacts with other interventions, such as cognitive training. Longitudinal randomized controlled trials are scarce, and few studies track long-term cognitive trajectories beyond 5-10 years post-surgery. Moreover, underrepresented populations, including racial and ethnic minorities and low-income groups, are rarely the focus of existing studies, despite being at increased risk for both cataracts and cognitive decline. Further research is needed to clarify optimal surgical timing, understand the mechanisms of cognitive benefit, and explore how integrating vision care with cognitive screening might offer synergistic benefits.

As the body of evidence grows, the potential role of cataract surgery as a modifiable factor in cognitive aging invites serious consideration among clinicians and policymakers. With dementia-related healthcare costs expected to rise exponentially and cataract surgery remaining a highly cost-effective intervention, these findings carry important implications for public health strategies targeting older adults [82,83]. The next section explores how these insights may inform clinical decision-making, early screening protocols, and population-level efforts to reduce the burden of cognitive impairment and dementia.

Clinical and public health implications

The current literature supports the integration of ophthalmologic evaluations into routine geriatric care. Primary care providers and Neurologists should collaborate with Ophthalmologists to ensure timely cataract detection and management in patients with or at risk for cognitive impairment. Visual assessments could serve as a low-cost, non-invasive tool for identifying older adults who might benefit not only from improved visual function but also potentially from reduced cognitive decline.

**Figure 3:**
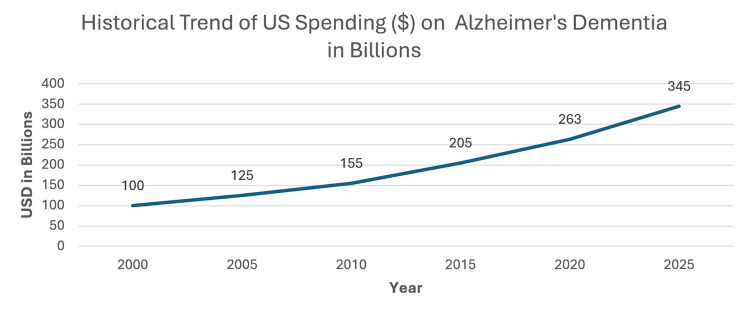
Historical Trend of US Spending ($) on Alzheimer’s Dementia in Billions Image Credits: Martena Grace and Amir A. Estil-las

Expanding access to cataract surgery, particularly in underserved and aging communities, would be a strategic intervention to promote healthy aging. Given that visual impairment is a modifiable risk factor, prioritizing vision correction aligns with dementia prevention initiatives, particularly as the global burden of cognitive disorders continues to climb. In 2025, dementia-related care in the United States is projected to cost $781 billion, and that figure is expected to reach $1 trillion by 2050 [84]. Figure 3 demonstrates the historical funding the US has invested in Alzheimer’s dementia-related care [85]. Prevention strategies like cataract surgery could reduce the severity or onset of dementia symptoms, potentially offsetting some of this economic burden.

However, several areas warrant further investigation. The precise biological mechanisms linking improved vision to cognitive preservation remain unclear. Additionally, more randomized controlled trials are needed to distinguish whether the cognitive improvements observed are direct effects of cataract surgery or indirect outcomes of enhanced sensory and social engagement.

Methods and materials

Search Strategy: to explore the factors contributing to the relationship between cataracts and dementia. We conducted a comprehensive literature review using Google Scholar, PubMed, and Scopus. Our search covered publications with the phrases “cataracts causing dementia”, “dementia and cataracts”, “visual impairment and dementia”, “cataract surgery and dementia”, “burden of cataracts”, “burden of dementia”, “treatment of dementia”, and “treatment of cataracts.”

Additionally, we employed the aid of a large language model (LLM), Chat GPT, to conduct a literature search with the previously mentioned phrases, and the inclusion of articles containing statistics detailing the economic burden and funding for cataracts and dementia.

Selection of studies: Our search query resulted in over 440 relevant manuscripts discussing cataracts and dementia. We excluded duplicate studies from Scopus and PubMed and review articles with limited data. Studies were included if they addressed hypotheses linking cataracts and dementia, pathophysiological overview of both cataracts and dementia, treatment outcomes for cataracts and dementia, statistical evidence regarding incidence, prevalence, and economic burden for cataracts and dementia, and studies with measurable clinical outcomes in the domains of cataracts and dementia.

The main focus was to compile a review discussing recent theories and advancements in the treatment and approach to the relationship between cataracts and dementia. There was an emphasis on discussing the burden of both cataracts and dementia independently on our society, and discussing the accessibility of treatment options and prevention strategies for cataracts and dementia. This approach sought to bring forth the existing knowledge and identify gaps in both research and clinical implementation concerning cataract and dementia.

## Conclusions

Cataracts and dementia are two of the most prevalent conditions affecting the aging population. While traditionally considered separate entities, one visual and the other cognitive, emerging evidence suggests a complex relationship between them. Visual impairment from cataracts may exacerbate cognitive decline through mechanisms involving sensory deprivation, social isolation, increased cognitive load, and alterations in circadian rhythm. Epidemiological and interventional studies support the idea that cataract surgery not only restores vision but may also blunt cognitive decline, offering a low-risk, high-impact strategy for dementia prevention.

Understanding the relationship between sensory and cognitive health opens new roads for clinical care and public health intervention. With the continuing rise in the global burden of dementia, prioritizing timely cataract management in aging individuals may prove to be a crucial component in broader cognitive health strategies. By restoring sight, we may also preserve the mind.
